# Evaluation of the effects of hydroalcoholic extract *of Berberis vulgaris* root **on the** **activity of liver enzymes in male** hypercholesterolemic rats

**Published:** 2012

**Authors:** Soheila Taheri, Ali Zarei, Saeed Changizi Ashtiyani, Azam Rezaei, Saeed Zaheiri

**Affiliations:** 1 *Department of Physiology, Arak University ofMedical Sciences, Arak, I. R. Iran*; 2 *Islamic Azad University of Damghan, Damghan, I. R. Iran*; 3 *Islamic Azad University of Arsanjan, Arsanjan, I. R. Iran*; 4 *Department of Biology, Payame Noor University of Abadeh, Abadeh, I. R. Iran*

## Abstract

**Objectives:** Hyperlipidemia can cause a variety of diseases such as atherosclerosis, diabetes, and fatty liver which is followed by increased liver enzymes. Since *Berberis vulgaris* (*B. vulgaris*) root possesses antioxidant properties, the present study was conducted to investigate the effect of its extract on the activity of liver enzymes in rats.

**Materials and Methods:** In this experimental study, sixty Wistar rats were selected and allocated to six groups of ten each. The control group received a normal diet and the sham group received a fatty diet while the other groups including experimental groups received a fatty diet and the alcoholic extract of* B. vulgaris* at minimum (75 mg/kg), moderate (150 mg/kg), and maximum (300 mg/kg) doses by intraperitoneal injection (i.p.) or oral atorvastatin (10 mg /kg) with a fatty diet. At the end of this 21-day period, blood samples were drawn and the levels of the intended factors were measured. Data were analyzed using SPSS software version 11.5.

**Results:** The comparison of the obtained results showed that the levels of alanine transaminase (ALT) and alkaline phosphatase (ALP) enzymes in the sham group that only received fatty food increased (p≤0.05), whereas in the treatment groups receiving *B. vulgaris *extract as well as in the group receiving Atorvastatin, these enzymes significantly decreased; however, no significant changes were observed in aspartate transaminase (AST) levels.

**Conclusion:** Noticing the antioxidant properties of *B. vulgaris* root extract and its effects on reducing the activity of liver enzymes, the extract of this plant can be a good choice for improving the function of liver.

## Introduction

Researchers with interest in natural products have intensified their efforts towards scientific evaluation of traditional medicines. Barberry has played a prominent role in herbal healing for more than 2500 years. *B. vulgaris* (European barberry)/(Jaundice berry)/(Ambarbaris) /(Barberry) is a shrub in the Berberidaceae family, native to central and southern Europe, northwest Africa, and western Asia. Seedless barberry (*B. vulgaris var. asperma*) is one of the most important medicinal fruit plants in the south of Khorasan province in Iran. Zereshk or Sereshk is the Persian name for the dried fruit of *B. vulgaris*, which is widely cultivated in Iran (Mohammadi et al., 2011 ▶). India's Ayurveda healers have used it for dysentery (Jellin et al., 2000 ▶).

During the early middle ages, European herbalists used it to treat liver and gallbladder ailments (Jellin et al., 2000 ▶). Russian healers used it for inflammations, high blood pressure, and abnormal uterine bleeding (Jellin et al., 2000 ▶). In folk medicine, European barberry root bark has been used for various conditions including liver dysfunction, gallbladder diseases, diarrhea, indigestion, and urinary tract diseases (Jellin et al., 2000 ▶).

The stem, root bark, and fruit of barberry contain isoquinoline alkaloids (e.g. berberine), which are the main active ingredients of barberry (Arayne et al., 2007 ▶). The bitter compounds in barberry, including alkaloids, stimulate digestive functions following meals (Arayne et al., 2007 ▶). Barberry and goldenseal (*Hydrastis canadensis*) have very similar therapeutic uses because both contain similar active substances (berberine alkaloids (Arayne et al., 2007 ▶)). These substances have been shown to combat infections and bacteria, stimulate the activity of the immune system, and lower fever (Arayne et al., 2007 ▶). Pharmacological studies suggest that these substances have antimicrobial and antiparasitic, anti-inflammatory, immune-stimulant, fever reducing, hypotensive (causing a reduction in blood pressure), sedative, anticonvulsant, and smooth muscle relaxant effects. Smooth muscles line the gastrointestinal tract; therefore, this last effect may help improve digestion and reduce stomach pain (Arayne et al., 2007 ▶).

Mammals’ cells possess enzymatic and non-enzymatic capability against the formation of bonds between them and free radicals. Non-enzymatic capability consists of beta-carotene, vitamin C, and vitamin E. The involved enzymes include superoxide dismutase, catalase, and glutathione peroxidase. In case the anti-oxidant defense mechanism is lost in the body, an increase in the formation of free radicals can cause cellular oxidative stress. Lipid peroxidation and oxidant stress increase in diabetes and hyperlipidemia (Kelly and Husband, 2003 ▶; Nazari et al., 2005 ▶). Recently, the inhibitory effect of *B. vulgaris* root extract on oxidative stress has been reported (Noori et al., 2004 ▶). Hyperlipidemia can also play an indirect role by activating the production of free radicals such as monocytes and neutrophils (Pourghassem et al., 2009 ▶). 

Dietary habits have a major effect on coronary risk factors and hyperlipidemia (Zarei et al., 2011 ▶; Heidarian et al., 2008 ▶). These diseases have developed across a wide range of developed and inderdeveloped societies. Although using lipid lowering anti-lipid chemical drugs such as lovastatin and atorovastatin can reduce hypercholesterolemia and cardiovascular diseases, such drugs have side effects (Zarei et al., 2011 ▶; Heidarian et al., 2008 ▶). Factors, such as patients’ dissatisfaction with chemical drugs, presence of side effects over long-term use and overuse, and financial burdens imposed on the patients, have led to an increased tendency towards alternative and traditional medicine. Using medicinal plants and fruits, in addition to decreasing health care costs in may societies, has had satisfactory results.

*B. vulgaris *is a medicinal plant that is commonly applied to the treatment of liver and biliary diseases in traditional medicine. *B. vulgaris* has secondary metabolites such as berberin, berlambine, oxyberberine, oxycanthine, chlorumamine, anthocyanine, bervulcine, lambertine, and magniflorine, which are frequently used in the pharmaceutical industry (Arayne et al., 2007 ▶; Mohammadi et al., 2011 ▶) and plays a major role in the treatment of gastrointestinal diseases, hemorrhages, gum inflammation, sore throat, biliary fevers, malaria, leishmaniasis, hepatitis, inflammation, diarrhea, and high blood cholesterol (Arayne et al., 2007 ▶; Mohammadi et al., 2011 ▶; Vrzal et al., 2005 ▶). Previous studies have shown that the aqueous extract of* B. vulgaris* fruit activates liver function and is effective in dissolving and possibly moderating blood cholesterol levels. Moreover, the extract of this plant reduces blood cholesterol and triglyceride levels (Farhadi, Gavadifar, 2008 ▶). In fact, *B. vulgaris* roots possess hypoglycemic properties (Golfaraz, Ahmad, 2008 ▶). Based on the studies done on alkaloids in* B. vulgaris*, such as berberin, anthocyanins, and its phenolic compounds, the antioxidants properties of the compounds present in this plant have been proven (Mohammadi et al., 2011 ▶; Hwong 2006 ▶; Motalleb, 2005 ▶).

Therefore, noticing the previous studies on the antioxidant properties of* B. vulgaris* and with regard to the hypoglycemic and triglyceride reducing effects of this plant and the relationship between these factors and liver enzymes, this study was carried out to investigate the effects of *B. vulgaris* root extract on the activity level of liver enzymes in hypercholesterolemic rats and to compare its effect with that of a hypoglycemic drug (atorvastatin) (Farhadi et al, 2008 ▶).

## Materials and Methods

This experimental study was done on sixty male rats supplied from the Razi Animal Breeding Center of Fars province. The rats were kept in standard conditions of temperature and light ad libitum. Animal care and handling were performed according to the guidelines set by the Iranian Ministry of Health and Medical Education for lab animals. Before launching the project, all of the rats were weighed to make sure that they are within the same range of weight (170±5 g).The rats were assorted into six groups of ten each in the following way:

A) Control group: The rats did not receive any vehicle or drugs and had a normal diet. B) Sham group: Hypercholesterolemic rats (2% cholesterol was added to their food to render them hypercholesterolemic) were daily administered 0.2 ml of normal saline as intraperitoneal injection (i.p.) for 21 days. C) Treatment group 1: Hypercholesterolemic rats were administered 75 mg/kg (minimum dose) of the alcoholic extract of *B. vulgaris* (i.p.) for 21 days. D) Treatment group 2: Hypercholesterolemic rats were administered 150 mg/kg (moderate dose) of the alcoholic extract of *B. vulgaris* (i.p.) for 21 days. E) Treatment group 3: Hypercholesterolemic rats were administered 300 mg/kg (maximum dose) of the alcoholic extract of *B. vulgaris* (i.p.) for 21 days. F) Atorvastatin group: In this group, 10 mg/kg of atorovastatin in the form of oral emulsion was administered to hypercholesterolemic rats through gavage for 21 days (Gosain S et al. 2010).

**Preparation of high cholesterol 2% food**

For preparing high cholesterol 2% food, 20 g of pure cholesterol powder (Fluka Chemika) was solved in 5 ml of heated olive oil and was mixed with 1 kg of the rats’ food. The food was kept in a refrigerator only for two days. (Rafati et al., 2006 ▶)

**Extraction method**

For preparation of the alcoholic extract of *B. vulgaris*, after obtaining the roots from the Shiraz University Herb Farm, Iran, and removing the impurities, 500 mg of the roots were milled and mixed with ethylic alcohol 98% at the ratio of 1:5. The mixture was kept in a capped container for 4 hours in lab conditions and was filtered by small and big filter papers. The filtered liquid was placed in water bath for solvent evaporation. Eventually, different doses (mg/kg bodyweight) of the extract (10 g per 100 g of the milled roots) were prepared by normal saline. All of the treatment groups received a fatty diet during the experiment. The experiment was carried out over a 21-day period and the injections were done through gavage every day at 9 a.m. as i.p. injections were made using insulin syringe and atorvastatin (10 mg/kg) (Shafa Pharmaceuticals Co., Iran) was administered as oral emulsion. After this period, mild anesthesia using ether was done for obtaining blood samples from heart to examine the concentration of plasma biochemical factors. After centrifuging blood at 3000 rounds per minute, serum samples were isolated and transferred to the lab for measuring the intended factors. The amount of liver enzymes contains: alanine transaminase (ALT), alkaline phosphatase (ALP), and aspartate transaminase (AST) were assessed through radio immunoassay (RIA) and Pars Azmoon kit using RIA1000 (USA). All obtained values are expressed as mean±SD and data analyses were done using SPSS ver. 11.5. The group's data means were compared by one-way analysis of variance and Tukey’s post hoc test. The level of significance was set at 0.05.

## Results

The comparison between the results of statistical analysis on the effects of *B. vulgaris *root extract and atorvastatin on the activity level of liver enzymes in hypercholestromic rats (Table 1) indicated that the amount of ALP in the sham group receiving a fatty diet had significantly increased in comparison with the control group, whereas changes in all of the treatment groups receiving the extract and the group receiving atorovastatin presented significant decreases compared with the sham group (p=0.00). 

**Table 1:** The comparison between effect of different doses of Berberis vulgaris (*B. vulgaris*) and atorvastatin on the parameters of liver activity in hypercholesterolemic rats

**Group** **Parameters**	**Control**	**Sham**	Atorvastatin	***B. vulgaris*** **Maximum Dose**	***B. vulgaris*** **Moderate Dose**	***B. vulgaris*** **Minimum Dose**
**Cholesterol**	85.00±0.98	97.25±3.13	80.62±2.02	76.12±5.11	86.00±3.95	86.00±5.35
**ALT(U/l)**	55.87±3.7	80.37±6.7	65.28±2.4	41.71±6.8 †# α	56.57±5.8	59.12±4.7
**AST(U/l)**	234.83±15.4	277.71±38 7	177.43±5.5	244.29±41.5	300.38±34.9	286.83±29.9
**ALP(U/l)**	1017.30±71.8	2400 ±44.5	673.62±12.1	914±10.6	1023.90±14.0	1027.16±10.3

† Comparison with the sham group,

* Comparison with the control group,

# Comparison with the atorovastatin group,

α Comparison between the minimum and maximum doses.

However, none of the treatment groups compared with one another and compared with the atorovastatin group presented significant changes. In terms of ALT, changes in the sham group were significantly higher than the control group, whereas its amounts in all of the treatment groups receiving B. vulgaris root extract as well as the atorovastatin group had significantly decreased. Moreover, the treatment group receiving the maximum dose in comparison with the minimum extract dose and the atorovastatin group showed a significant decrease (p=0.001). 

This, in fact, indicates that the effect of the maximum dose on the amount of ALP decrease is much stronger than other doses and even atorovastatin. Regarding AST, changes in the sham group are not more significant in comparison with the control group and its amounts in the treatment group does not indicate significant changes (p=0.090). There were not shown any significant changes in liver enzymes between moderate dose of B. vulgaris extract with minimum and maximum doses (Table 1).

## Discussion

The comparison of the results of statistical analyses showed that ALT and ALP enzymes amounts in the sham group that only received a fatty diet had increased, whereas these enzymes in the treatment groups receiving B. vulgaris extract and the atorovastatin group had decreased; however, no significant changes were observed in AST. Non-alcoholic fatty liver disease (NAFLD) is an insulin-resistant pathogenic factor in type II diabetes. Liver enzymes levels in circulation, including ALT, AST, and gamma glutamyl transferase, on the other hand, in asymptomatic with NAFLD are high.

Following reduction in the amount of insulin the metabolism of acids in the liver is disrupted and eventually fatty liver is resulted which is followed by increases in ALT, AST, and ALP levels (Bush 1991 ▶; Jelodar, Nazifi, 1997 ▶). Normally, following reduction in serum cholesterol, liver lipidosis decreases and the activity of liver enzymes gets significantly decreased (Jelodar, Nazifi, 1997 ▶; Yu, Keeffe, 2003 ▶). In this study, fatty liver diseases are defined as the deposition of fat (mainly neutral fats such as triglyceride) in the liver which are usually accompanied by increased liver enzymes (ALT and AST) that may result in liver inflammation. This issue can cause liver hardening and scarring that result in liver cirrhosis. With progression of the disease, scarred and hardened tissues are develop around liver cells and gradually cause nodules and cysts in liver which, in turn, by creating pressure on biliary ducts , lead to obstruction in these ducts, and, eventually, increased bilirubin and cholesterol.

Free radicals destroy cell membranes, such as hepatic cells, which result in increasing liver enzymes. This causes the entrance of enzymes that are normally located inside cell cytosol into blood circulation and increased activity of these enzymes indicates the degree and type of liver damage (Shariati and Zarei, 2006 ▶). Therefore, noticing the aforementioned studies and the findings of the present study, increase in the amount of ALT in the sham group that only received fatty food is predictable. *B. vulgaris* has anti-oxidant properties and the berberin in this plant effectively reduces lipid peroxidation with its anti-oxidant properties. This way, it protects antioxidant enzymes, against damage due to oxygen free radicals and peroxide hydrogen (Thirupurasundari et al., 2009 ▶; Ziai et al., 2010 ▶). Antioxidant activities of the ethanolic extracts of roots, twigs, and leaves of common barberry (*B.* *vulgaris* L.) were studied. All the extracts were found to possess some radical-scavenging and antioxidant activities (Zovko et al., 2010).

In their analysis of *B. vulgaris* root extract, Tomosaka *et al.* found three phenolic compounds with antioxidant and anti-inflammatory properties (Tomosaka et al., 2008). Therefore, considering the anti-oxidant properties of *B. vulgaris*, reduction in the activity of liver enzymes in the treatment groups receiving the plant extract is predictable.

Increase in HDL-C lipoprotein results in reduction of cholesterol level and improvement of liver function (Hu et al., 2003 ▶). *B. vulgaris* plays a significant role in reducing cholesterol (Arayne et al., 2007 ▶; Mohammadi et al., 2011; ▶ Vrzal et al., 2005 ▶) and its extract decreases cholesterol and triglyceride levels (Farhadi et al., 2008 ▶); therefore, it can be applied to the treatment of liver diseases. Noticing this cholesterol-reducing property which, in turn, decreases liver lipidosis that has a significant relationship with reduction of liver enzymes activity, in the present study, the decrease in liver enzymes in the treatment groups receiving the extract seems logical. There is a direct relationship between the amount of lipids and leptin, whereas there is an indirect relationship between the level of thyroid hormones and lipids. Furthermore, with increase in the amount of lipids and their deposition in the liver, the level of liver enzymes increases (Bush, 1991 ▶; Jelodar, Nazifi, 1997 ▶; Saeb et al, 2010 ▶; Tohidi, 2007 ▶).

By comparing the aqueous and alcoholic root extracts of *B. vulgaris*, Shahid *et al*. demonstrated that both extracts possess significant anti-inflammatory effects. The alcoholic extracts possibly play their role through blocking mediators which are released in the delaying phase of inflammation such as prostaglandin and the aqueous extract through blocking mediators, such as bradykinin, histamine, and serotonin, which are released in the primary phase and partly blocking the mediators in the delaying phase, such as prostaglandin (Shahid et al., 2009 ▶).

As it was mentioned before, increased lipids and liver inflammation result in an increase in liver enzymes and since* B. vulgaris* has anti-inflammatory properties and reduces cholesterol level, the decrease in liver enzymes seems reasonable (Noori et al., 2004 ▶). Ivanovska et al.’s study on the effective substances in the alcoholic root extract of *B. vulgaris* indicated the presence of effective substances such as berberine and oxyacanthine with anti-inflammatory effects which their degree of influence depends, to a great extent, on their duration of use (Arayne et al., 2007 ▶). Fallah Huseini et al’s study on the effects of *B. vulgaris *L. Root extracts on carbon tetrachloride induced liver toxicity shows that *B. vulgaris *root extracts prevented CCI_4_ induced hepatotoxicity in rats (Fallah Huseini et al, 2010 ▶). Hyperlipidemia also triggers the production of free radicals (Heidarian et al., 2008 ▶; Sharma et al., 2009 ▶). In addition, in patients with liver toxicity, active metabolites formed through P_450 _increase and result in liver necrosis. Here, the toxic amounts depend on glutathione level (Chiou, Tzeng, 2000 ▶; Callberg, Mannervik, 1985 ▶). TNF-α and IL-6, on the other hand, are inflammatory cytokines that have a close relationship with leptin and lipid percentage (Boghrabadiet al., 2009 ▶).

Alkaline phosphatase is a trans-peptidase that increases in bone and liver diseases. Research has shown that phenolic compounds in medicinal plants can prevent the toxic effects of drugs on the liver and cause the release of glutamic pyruvic transaminase (SGPT) and alkaline phosphatase into blood (Nazari et al., 2005 ▶). 

## Conclusions

The findings of the present study show that alcoholic extract of *B. vulgaris* root could play an important role in lowering ALP and ALT. However, more studies are needed to determine the underlying mechanisms.
